# Early neutropenia on day 8 treated with adjuvant Docetaxel-based chemotherapy in early breast cancer patients: Putative mechanisms within the neutrophil pool system

**DOI:** 10.1371/journal.pone.0215576

**Published:** 2019-04-18

**Authors:** Yoshihiko Furuya

**Affiliations:** Department of Surgery, Saiseikai Osaka Nakatsu Hospital, Osaka, Japan; Istituto di Ricovero e Cura a Carattere Scientifico Centro di Riferimento Oncologico della Basilicata, ITALY

## Abstract

Most chemotherapy regimens cause neutropenic nadirs between days 10 and 14, and administration of granulocyte colony-stimulating factor (G-CSF) support relies on this timing. In docetaxel (DOC)-based chemotherapy, the frequency of febrile neutropenia (FN) and the G-CSF dose administered varied greatly between studies. Our study goal was to forecast the necessary dose of G-CSF by comparing day 8 neutropenia with putative changes within the neutrophil pool. We conducted a retrospective observational analysis of 242 early breast cancer patients who had received adjuvant DOC-based chemotherapy (DOC group) compared with 43 patients who had received FEC chemotherapy (FEC group). Patients who were given a standard dose and had a blood test on day 8 in the 1^**st**^ cycle were eligible. In the DOC group, patients routinely received prophylactic administration of G-CSF (150 μg/body) on day 3 and received additional G-CSF based on a blood test on day 8. Results of the day 8 blood test showed that severe neutropenia (<500/mm^3^, average 494/mm^3^) was observed in 152 out of 242 (62.8%) patients in the DOC group, while in the FEC group (n = 43), neutropenia was ambiguous (average 1,741/mm^3^). In the FEC group, 9 out of 43 patients (20.9%) and in the DOC group, 27 out of 242 patients (11.1%) experienced FN. In the DOC group, day 8 neutropenia was predictive for FN in a logistic regression model (OR 0.79 [95% CI: 0.655–0.952], p = 0.013). Among 214 patients under 70 years old, the planned chemotherapy cycle was completed in 190 (88.8%) patients who also received the maximum dose of G-CSF (150 μg/body) four times, while 23 patients could not complete the planned chemotherapy cycle, but only five because of FN-related complications. Patients treated with DOC should be treated for primary prophylaxis with G-CSF support at an earlier time starting with a relatively small dose.

## Introduction

Severe neutropenia caused by myelosuppressive chemotherapy predisposes patients to serious infections. Febrile neutropenia (FN), generally defined as fever with grade 3/4 neutropenia (neutrophil count <1000/mm^3^ and <500/mm^3^, respectively) [1], is associated with substantial morbidity and represents an oncologic emergency [2, 3].

The neutrophil is a critical effector cell in a host’s immune defense against microbial infection, and its lifespan is regulated by various pathogens and host-derived substances [4, 5]. The neutrophil can move freely through the walls of veins and into the tissues to immediately attack all invading foreign substances. Neutrophil homeostasis is maintained by a fine balance between granulopoiesis, bone marrow storage and release, and intravascular margination. The neutrophil population in the bone marrow can be subdivided into three pools: the stem cell pool, the mitotic pool, and the postmitotic pool. The mitotic pool, which is sensitive to myelosuppressive chemotherapeutic drugs, refers to committed granulocytic progenitor cells that are undergoing proliferation and differentiation. After release into the bloodstream, a proportion of neutrophils can be mobilized back into this freely circulating pool; this recoverable portion of neutrophils is termed the marginated pool.

Primary prophylaxis with G-CSF, used as daily filgrastim or once-per-cycle pegfilgrastim, reduces the severity and duration of chemotherapy-induced neutropenia and the consequent risk of FN, and plays an increasingly broad role in supporting the delivery of myelosuppressive chemotherapy [6–10]. To counter the effects of highly myelosuppressive chemotherapy, pegfilgrastim has been shown to lower FN rates by approximately one third compared with daily G-CSF given for 10–14 days [11]. Therefore, pegfilgrastim is the most commonly prescribed primary prophylaxis for high risk of FN caused by myelosuppressive chemotherapy [12].

Data from the US Oncology Adjuvant Trial 9735 has shown that DOC and cyclophosphamide (TC) improved overall survival when compared with doxorubicin and cyclophosphamide in early stage breast cancer. Despite 61% grade 3/4 neutropenia in the TC arm, only 5% of patients developed FN without primary prophylactic G-CSF [13]. Although prophylactic antibiotics reduce morbidity by preventing the spread of infecting pathogens, the prevailing determinant for the onset of infection remains circulating neutrophils. The worldwide adoption of this protocol yielded several reports on substantially higher rates of FN events and therefore, recommended primary prophylaxis with G-CSF. Without routine prophylactic G-CSF administration, the highest rates of FN events were found in Japanese (28.3%) and Canadian (33%) studies [14, 15]. Pegfilgrastim, equivalent to over 10 doses of daily 300 μg/body filgrastim, lowered the incidence of FN in breast cancer patients receiving adjuvant TC chemotherapy [16]. Recently, fewer doses of only 2 or 3 times 300 μg/body filgrastim were enough as prophylactic G-CSF, which resulted in less side effects, such as bone pain and incidence of fever [17, 18].

Regarding timing of the appearance of the neutrophil nadir, DOC monotherapy has been reported to result in severe neutropenia several days earlier than with conventional chemotherapy, with a median time to neutropenic nadir of 7 days [19]. There seems to be a discrepancy in DOC-based regimens between severity of neutropenia and subsequent FN. Having a high rate and grade of neutropenia several days earlier did not match with the rate of consequent FN, which could be prevented by administering a relatively small amount of prophylactic G-CSF. To clarify this, we conducted a retrospective observational study to determine if DOC induced early neutropenia and discussed its relevance to the neutrophil reserve pool.

## Patients and methods

The study was approved by the institutional review board of the Saiseikai Osaka Nakatsu Hospital and written informed consent was obtained from all patients prior to surgery or primary chemotherapy. This retrospective analysis included all consecutive patients in an electronic database who were treated with TC, FEC-DOC, and FEC chemotherapy with standard doses for breast cancer at the Saiseikai Osaka Nakatsu Hospital, Osaka, Japan from May 2008 to March 2018. The regimen employed TC (DOC 75 mg/m^2^ and cyclophosphamide 600 mg/m^2^ administered i.v. every 3 weeks), FEC (fluorouracil 500 mg/m^2^, epirubicin 100 mg/m^2^, and cyclophosphamide 500 mg/m^2^ administered i.v. every 3 weeks), and DOC (100 mg/m^2^ i.v. every 3 weeks after completion of three cycles of FEC), as described in the NCCN Guidelines. Doses prescribed were within 5% of the calculated doses based on body surface area.

Eligibility criteria were as follows: patients who received at least 1 cycle of a standard dose regimen of TC, DOC, or FEC with white blood cell differentiation count data on day 8 in the first chemotherapy cycle, no previous chemotherapy or radiotherapy, and histologically proven breast cancer without distant disease. Along with the DOC-containing regimen, prophylactic filgrastim was routinely administered subcutaneously at a dose of 150 μg/body on day 3, and on the basis of the blood test on day 8, patients could receive an additional one or two doses of filgrastim at a dose of 150 μg/body. In this study, chemotherapy doses were calculated based on body surface area, but G-CSF doses and dexamethasone premedication were given at half of what was stated in the literature because of the low body weight of the patients in this study (body weight; median 54kg, range 36-90kg). Estrogen receptor (ER) status was determined using immunohistochemistry (IHC); and HER2/neu status was determined using IHC and/or fluorescence in situ hybridization, which were performed at BML Inc. (Tokyo, Japan). Concomitant use of trastuzumab with TC or DOC monotherapy was administered to 30 out of 31 patients whose tumors were HER2-positive.

### Statistical analysis

Group comparisons for blood count variables were carried out using an unpaired Student’s t-test. A logistic regression model was used to estimate the FN factors. Chi-square tests were used to determine the significance of difference between categorical variables. A two-tailed p value of less than 0.05 was considered statistically significant and all statistical analyses were performed using the software package IBM SPSS Statistics v21.

## Results

### Study population

In total, 255 consecutively treated patients were identified in this retrospective study within the study timeframe. Five patients were excluded (two patients with distant disease, one patient with extravasation, and two patients who had received chemotherapy previously). Patient demographics and tumor characteristics for the TC, FEC-DOC, and FEC treated groups are shown in Table 1. Two hundred and seven patients treated with TC were included in the DOC group, 35 patients treated with FEC-DOC were analyzed in both the FEC and DOC groups, and eight patients were treated with the FEC regimen only. Therefore, 242 cases of chemotherapy administration were defined as the DOC group and 43 cases were defined as the FEC group. Median (range) age was 57 (22–80), 57 (35–76), and 55 (47–78) years in the TC, FEC-DOC, and FEC groups, respectively.

**Table 1:** Patient and tumor characteristics (n = 250).

	TC n = 207	FEC DOC n = 35	FEC, n = 8
Age Median (range), years	57 (22–80)	57 (35–76)	55 (44–78)
Estrogen receptor			
Positive	192	10	1
Negative	15	25	7
HER2 Status			
Positive	28	3	0
Negative	179	32	8
N stage			
N0	89	12	5
N1	70	6	1
N2,3	43	17	2
NA	5	0	0
T stage			
T1	95	7	4
T2	92	18	3
T3	13	4	1
LN or skin rec	7	3	0
Inflammatory	0	3	0

Patient demographics and tumor characteristics for the TC, FEC-DOC, and FEC treated groups.

HER2 positive cases were defined as IHC 3+ or if IHC 2+, FISH with amplification ratio >2.0 *TC* docetaxel/cyclophosphamide. *FEC* fluorouracil/epirubicin /cyclophosphamide. *DOC* docetaxel. *NA* not assessed.

### Chemotherapy and G-CSF exposure

In the DOC group during the first cycle, all 242 patients received 150 μg/body of filgrastim administered on day 3. Administration of filgrastim (150 μg/body) was determined according to either day 8 neutrophil counts or if FN had already occurred; therefore, filgrastim was administered once in 40 patients, twice in 177 patients, and three or more times in 25 patients during the 1^st^ cycle. Among 214 patients who were under 70 years old, 190 patients completed the planned cycle with the planned dose intensity. In this group, filgrastim (150 μg/body) was administered once in 38 patients, twice in 138 patients, and three or more times in 14 patients during the 2^nd^ cycle.

### Day 8 neutrophil counts in TC, DOC, and FEC-treated patients (Table 2 and Fig 1)

**Fig 1:**
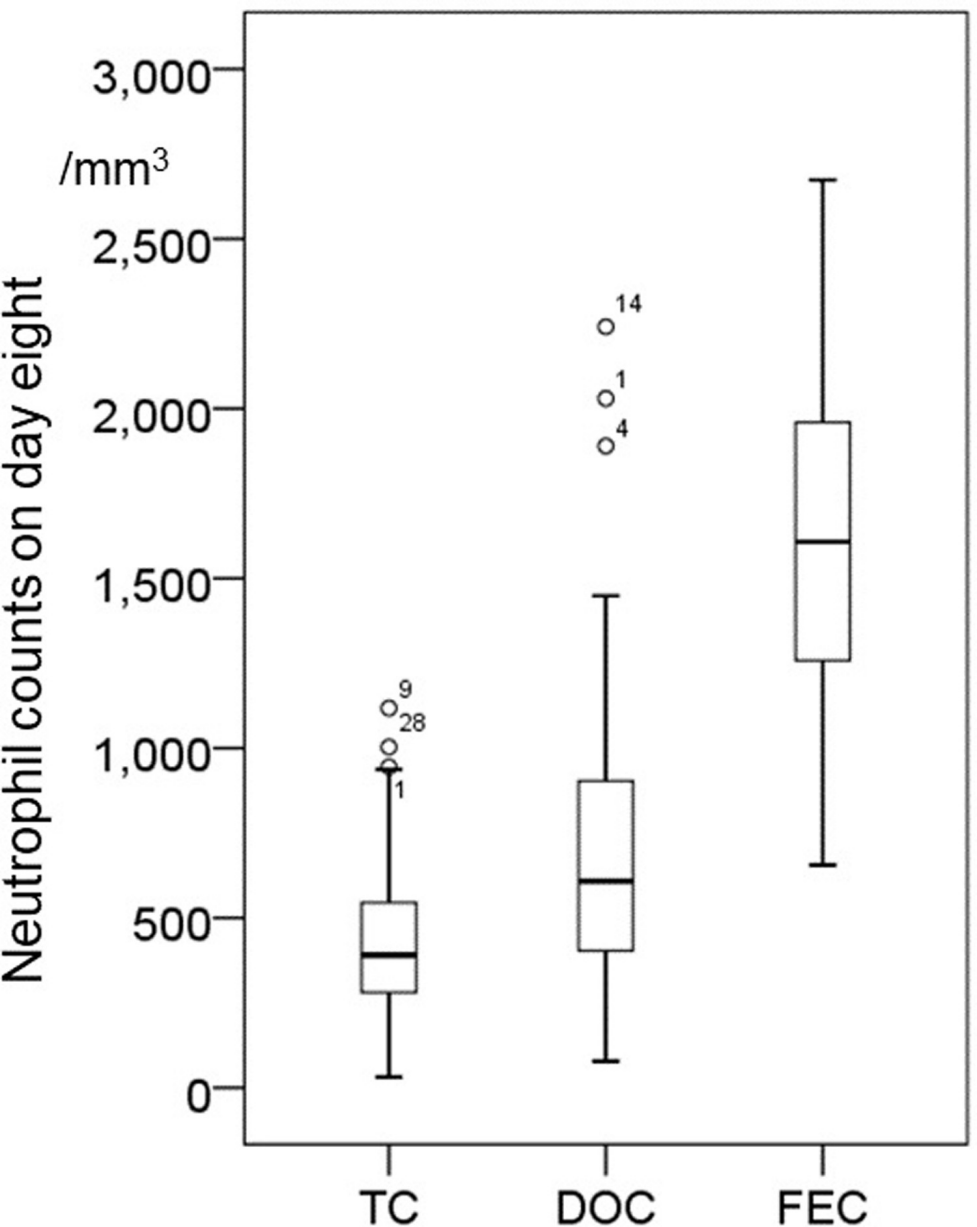
Absolute neutrophil count on day 8 in TC, DOC and FEC-treated patients. Box-plot diagram of absolute neutrophil count on day 8 in TC-treated, DOC-treated and FEC-treated patients.

Peripheral neutrophil counts on day 8 are shown in Table 2 and Fig 1. Profound neutropenia <100/mm^3^ was observed in 12 TC-treated patients (average 452/mm^3^) and one DOC-treated patient (average 749/mm^3^), while no FEC-treated patient (average 1,740/mm^3^) had neutropenia (<500/mm^3^, with the lowest being 656/mm^3^). In DOC group grade 4 neutropenia (<500/mm^3^) was observed in 152 out of 242 patients (62.8%) on day 8. A significant difference was observed in neutrophil counts on day 8 between the FEC and DOC groups.

**Table 2:** Occurrence of febrile neutropenia and neutropenia grade on day 8.

	TC n = 207	DOC (after 3 FEC) n = 35	FEC n = 43
FN	24 (11.6%)	3 (8.6%)	9 (20.9%)
Day 8 neutropenia			
Grade 2	9	5	16
Grade 3	61	11	4
Grade 4	136	16	0
Neutrophil count			
<100/mm^3^	12	1	0

Patient demographics of febrile neutropenia and neutropenia grade on day 8 in the first chemotherapy cycle for the TC, FEC-DOC, and FEC treated groups.

Neutropenia grade (CTCAE) absolute neutrophil counts; grade2 <1500–1000/mm^3^, grade3 <1000–500/mm^3^, grade4 <500/mm^3^. *FN* febrile neutropenia.

### Early FN occurrence in the DOC group

**Fig 2:**
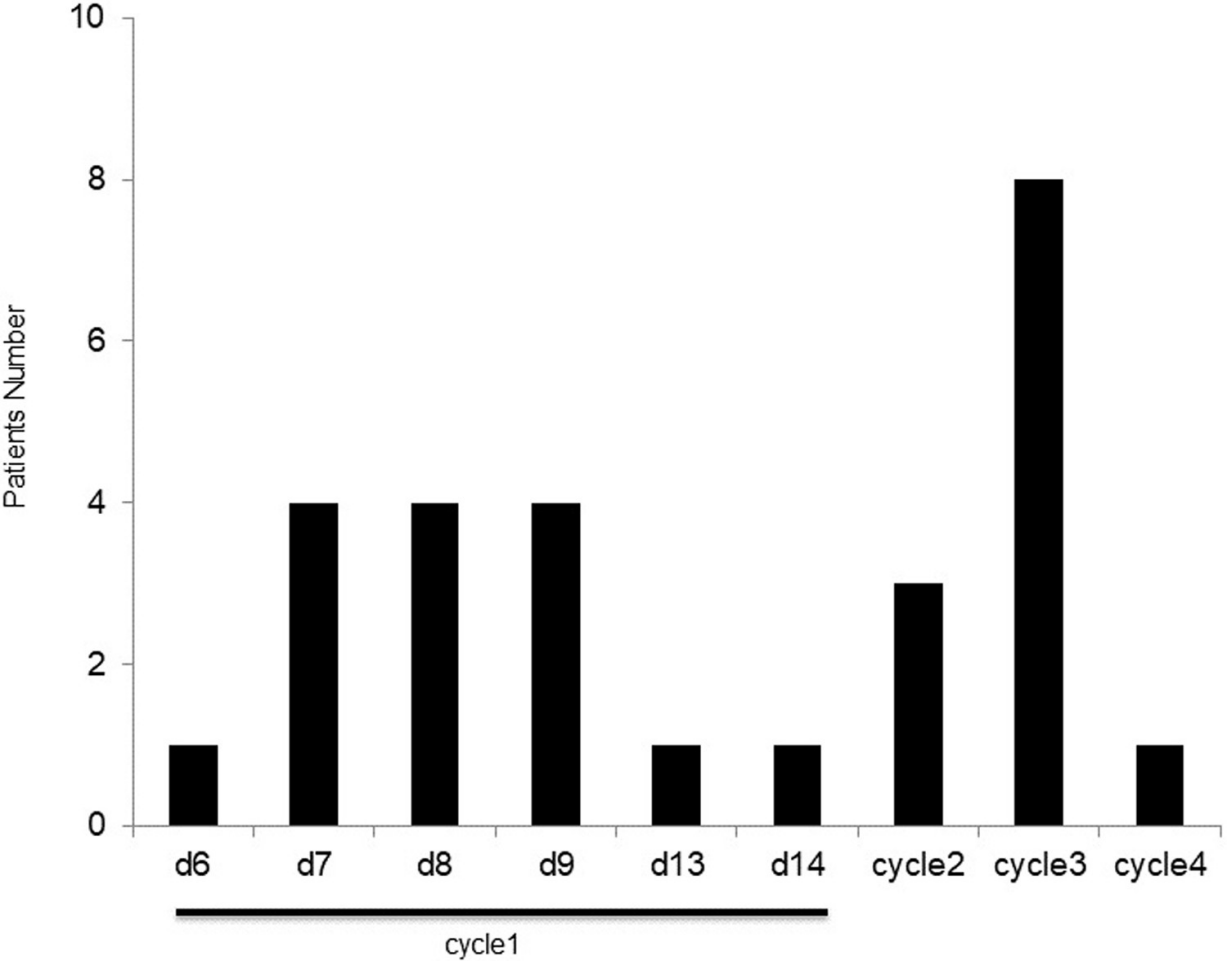
The first febrile neutropenia in DOC group. The bars represent the numbers of patients experienced the first occurrence time of febrile neutropenia in DOC group.

Among 27 patients in the DOC group, including two patients over 70 years old, 15 patients developed FN during the 1^st^ cycle, and the time of occurrence of FN was plotted on a bar graph (Fig 2). Seven patients experienced FN prior to the day 8 blood test. It is unlikely that fever observed on day 8 or 9 was caused by prophylactic filgrastim treatment because fever had not been observed in patients given the same dose of filgrastim on day 3.

### Occurrence of FN in DOC and FEC-treated patients: Neutrophil count on day 8 versus age (Fig 3)

**Fig 3:**
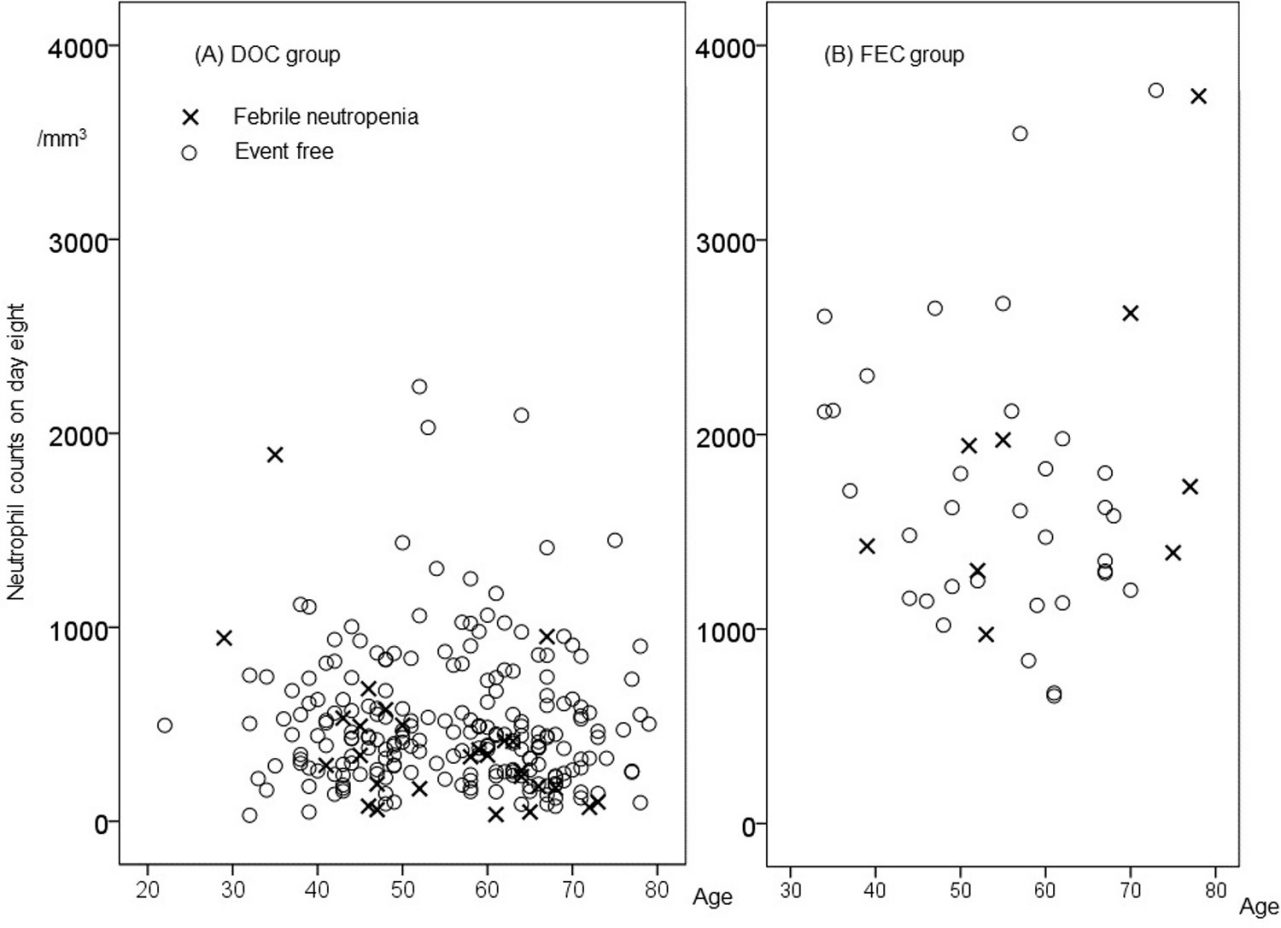
Febrile neutropenia and neutrophil counts on day 8 versus age. (A) in DOC group and (B) in FEC group, a scatter plot of age versus neutrophil count on day 8 is shown, patients experienced FN during a planned cycle were shown in punctuation mark (x) and who did not open circle (o).

A scatter plot of age versus neutrophil count on day 8 in both DOC and FEC-treated groups is shown in Fig 3. The FN rate was higher in the FEC group (20.9%, 9 out of 43 patients) compared with the DOC group (11.1%, 27 out of 242 patients); however, statistical significance was not reached (p = 0.075). Patients experienced FN during a planned cycle were shown in punctuation mark and who did not open circle. Age did not affect FN in either group.

### Risk factors predisposing patients to FN in the DOC group

Regression analysis was performed to determine which factor among age, and neutrophil, lymphocyte, and monocyte counts on days 1 and 8, was associated with a higher risk of FN. In the DOC-treated group, only day 8 neutrophil counts were significantly associated with neutropenic infection in the logistic regression model (OR 0.79 [95% CI: 0.655–0.952], p = 0.013). Patients with neutrophil counts of <500/mm^3^ were more likely to contract an infectious disease than those with neutrophil counts >500/mm^3^ (RR = 1.32, 95% CI 1.07–1.62). In the FEC group, none of the above factors were predictive of FN in the logistic regression model.

### Other toxicities in the DOC group

Among 214 patients who were under 70 years old, the planned cycle of the DOC-containing regimen was completed in 190 (88.8%) of them. In this group, 24 patients could not complete the planned cycle: five because of FN-related complications, eight because of severe allergic diseases including skin rashes, and 11 because of other reasons. In this cohort, no patients died from chemotherapy-related complications.

### Changes in baseline neutrophil counts by oral dexamethasone pretreatment in TC treated patients (Fig 4)

**Fig 4:**
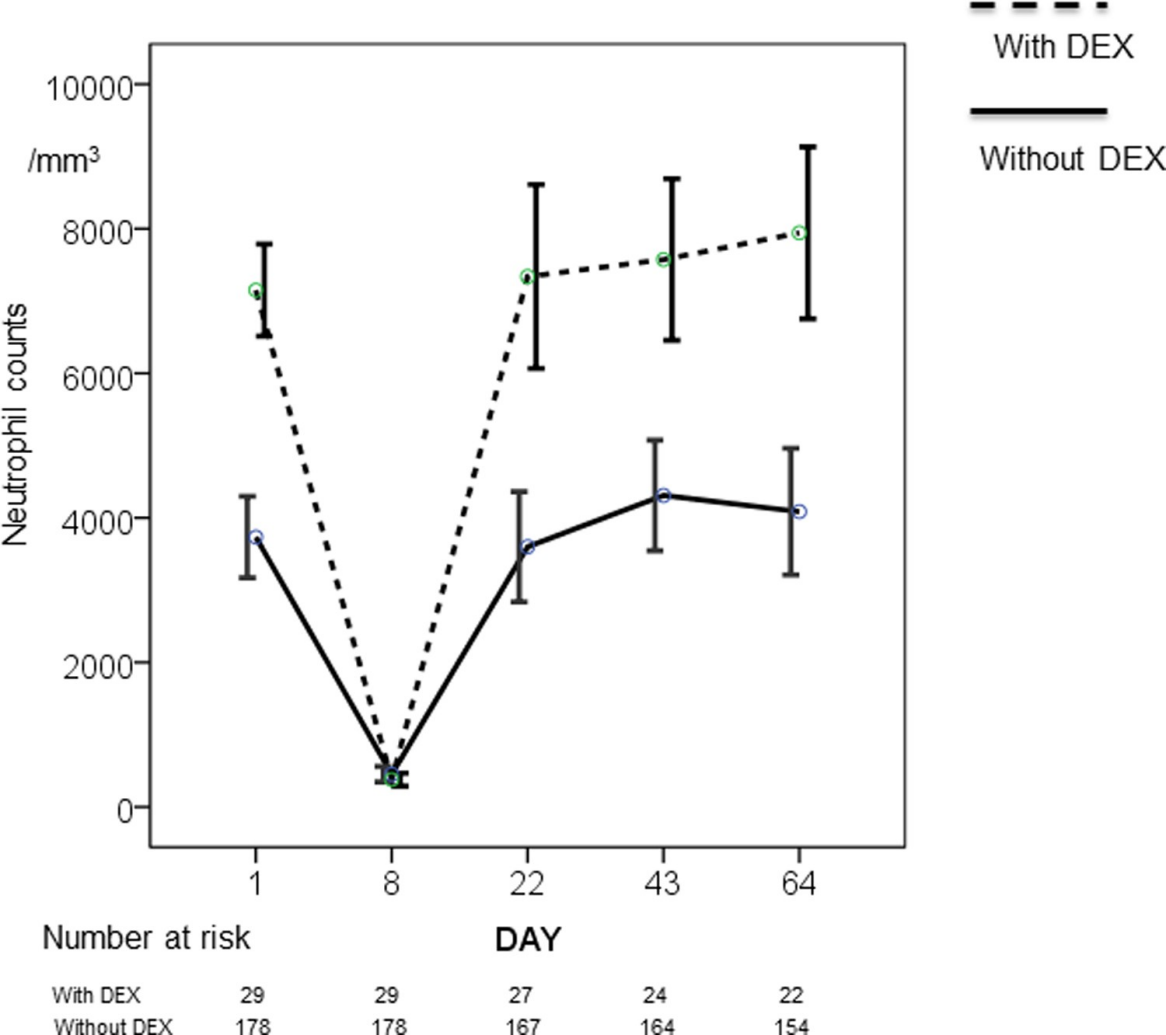
Changes in neutrophil counts by oral dexamethasone pretreatment in TC treated patients. Solid black line represents the absolute neutrophil counts without premedication oral dexamethasone and dashed line represent with oral dexamethasone premedication in TC treated patients.

Premedication for fluid retention was specified for all patients in the DOC-treated group and consisted of oral dexamethasone 4 mg, given 24, 12, and 1–2 hours before DOC infusion, routinely administered to patients from July 2016. Only 29 patients were premedicated in the TC group. Premedication with dexamethasone the day before TC chemotherapy resulted in a statistically significant 1.76–1.96 fold increase in neutrophil counts; however, day 8 neutropenia was observed in patients regardless of oral dexamethasone premedication (Fig 4).

## Discussion

The semi-mechanistic models for neutropenia generally used consist of three compartments [20]. Anticancer drugs affect each compartment and evoke a change in duration of each compartment and its respective interactions. The first is a proliferating compartment that is sensitive to chemotherapy drugs. Destruction of neutrophil progenitors in the bone marrow, known as the most rapidly proliferating cells in the body by a chemotherapy drug, is considered diagnostic of antiproliferative activity. A proliferating compartment sensitive to a chemotherapeutic agent then forms the hematopoietic niche. The second is a transit compartment that represents maturation. Postmitotic bone marrow neutrophils constitute 95% of the neutrophils in the body and this reserve is easily mobilized and recruited rapidly to sites of infection [21, 22]. Transit compartments predict the time delay that mimics the maturation chain in the bone marrow. Studies in healthy volunteers employing tritiated thymidine have shown that postmitotic transit time is ~6.5 days [23]. When recombinant G-CSF is administered, this is reduced to 2.9 days [24]. These durations of transit time between compartments have not been investigated in chemotherapy-treated patients in whom DNA damage to neutrophil progenitor cells can trigger a stop at the G2 to M-phase transition in the cell cycle. The third is a compartment of circulating mature neutrophils. Mature neutrophils had been thought to be remarkably short-lived with a circulating half-life of 6–8 h, but recent studies indicated that their lifespan was in the order of several days [25]. Neutrophil progenitors are diversely differentiated and their fate and lifespan are also known to vary.

In the semi-mechanistic model for neutropenia, the longer period of postmitotic transit time, increasing from 6.5 to 10–14 days, was explained by the differential distribution of the drug to the bone marrow, resulting in a lengthened period of transit time, mainly within the period of the proliferating compartment [20]. In the clinic, 10–14 days is the actual functional postmitotic transit time in chemotherapy-treated patients, and DOC accelerates the early appearance of the nadir because of a different mechanism, which might work within the reserve system of neutrophils. Two hypotheses could be considered. One is that impairment of the mobilization of the neutrophil reserve in the bone marrow occurs. The second is that part of the postmitotic neutrophil reserve at various maturity stages is directly damaged by DOC.

DOC, which belongs to the class of tubulin inhibitors, works by impairing microtubule dynamics, resulting in cell cycle alterations and cell death. Mature neutrophils in the bone marrow migrate across the bone marrow endothelium through tight-fitting pores by a unique process of transcellular migration. The neutrophil's microtubules were reported to play a different role, because microtubule disruption induces neutrophil polarity and impairs interpretation of chemoattractant gradients [26]. The possible mechanism that impairment of microtubule dynamics in neutrophils resulted in the impairment of the mobilization of the neutrophil reserve in the bone marrow was supported by data with other tubulin inhibitors in which an early neutropenia nadir has also been reported. Depending on the type of anticancer agent, the period until leukocyte depletion was reported to vary. Generally, it is around 10–14 days in the case of doxorubicin, carboplatin, etoposide, and cyclophosphamide. Conversely, drugs such as DOC, vincristine, and vinorelbine caused neutropenia on day 7 or earlier [27, 28]. Vincristine and vinorelbine are also tubulin inhibitors. The second hypothesis (see above), whereby DOC directly damages postmitotic neutrophils, is thought to be unlikely. The main role of the neutrophil is to engulf and destroy foreign material through phagocytosis [29]. Most neutrophils undergo programmed cell death after phagocytosing 5–25 bacteria. The pathogen undergoes phagocytosis and then degradation due to lysosomal activation in the neutrophil [30]. Phagosome fusion with the lysosome contributes to the killing and digestion of phagocytosed microorganisms. Though DOC evokes cancer cell death, concomitant activation of the lysosomal pathway and increased autophagy has been reported [31–33], although there have been no reports that mature neutrophils are damaged via such mechanisms by DOC.

From our results, the possibility of the latter mechanism cannot be ruled out. DOC-induced edema cannot be prevented with high dose venous infusions of dexamethasone immediately before DOC administration. DOC-related fluid retention is cumulative, reversible, and non-lethal, and severe symptoms occur in only 5%–6% of patients [34]; the mechanism has been reported to be a consequence of capillary protein leakage [35]. Premedication with oral dexamethasone the day before chemotherapy is started is regarded as the only reliable method of preventing fluid retention [36, 37]. Our results (Fig 4) show that oral dexamethasone premedication doubled baseline neutrophil counts and appeared to fully mobilize the marginated neutrophils because the fraction of marginated cells differed somewhat between experiments and was approximately 50% for neutrophils [38]. If mobilization of marginated neutrophils with histotoxic capacity is a prevention mechanism of fluid retention, administration of oral dexamethasone may not only prevent fluid retention but may also lead to the prevention of lymphedema in breast cancer patients and should be administered in all cases.

If DOC changes neutrophil reserves, baseline neutrophil counts might be considered as a reference instead of an inclusion criterion for administration of DOC-containing chemotherapy. Our data suggested that baseline neutrophil counts were not related to FN in DOC-treated patients. In other word, baseline neutrophil counts have been used clinically as reflecting the ability of granulopoiesis but may reflect factors as indicating a volume of the reserved postmitotic neutrophil pool, which is active till the nadir occurs. If this is correct then it might not be necessary to take an extra peripheral vein blood sample, nor would it be necessary to wait for the results of the blood test, including white cell differentiation counts. Further, with common anticancer drugs, dose reduction or delaying administration was sometimes recommended to restore progenitor cells. In the DOC group, it appears that there was less progenitor damage than the observed neutropenia severity; therefore, it might be worthwhile to prioritize dose intensity. Neutropenia caused by any mechanism can be lethal if the infection is worsened by the condition of the patient, the strength of the pathogen, or the patient’s susceptibility to antibiotics. G-CSF is the predominant factor regulating the neutrophil life cycle by increasing cell proliferation, survival, differentiation, and trafficking/mobilization.

There are retrospective data to suggest that patients who experience at least some degree of neutropenia while on their adjuvant chemotherapy may have an improved survival. In times when there were less chemotherapy options, several studies indicated that chemotherapy induced neutropenia predicts better outcomes in breast cancer those who had received adjuvant treatment with cyclophosphamide, methotrexate, and 5-fluorouracil (CMF) [39, 40] or cyclophosphamide, epirubicin, and fluorouracil (CEF) [41]. In modern days, patients can have a choice from several anticancer agents, therefore, in addition to the shortage of observation period, the subset population of this study became insufficient to study prognosis. Anticancer agents have various mechanisms of action, so validation of putative mechanism may be useful in studying individual differences in treatment outcome and toxicity.

Our study was hypothesis-generating and provided information stating that a relatively small amount of G-CSF should be administered early to DOC-treated patients. The hypothesis that DOC causes changes to the neutrophil pool system in addition to myelosuppression can explain our results and fits with results from numerous papers published previously [17–19]. To improve the actual clinical protocol, further study is warranted.

## Supporting information

S1 Dataset Raw data of neutrophil, lymphocyte and monocyte counts and patient characteristics. *ANC* absolute neutrophil count, *ALC* absolute lymphocyte count and *AMC* absolute monocyte count, *dex* dexamethasone pretreated.
(XLSX)
